# Development and Validation of a Self-Report Measure of Existential Well-Being

**DOI:** 10.2174/0117450179366317250410071321

**Published:** 2025-05-05

**Authors:** Angelo Picardi, Antonella Filastro

**Affiliations:** 1 Centre for Behavioural Sciences and Mental Health, Italian National Institute of Health, Rome, Italy; 2 Institute for Humanistic and Existential Psychotherapy (IPUE), Rome, Italy

**Keywords:** Psychotherapy, Existential philosophy, Wellbeing, Assessment, Validity, Reliability

## Abstract

**Background:**

Research in the field of existential psychotherapy has mainly relied on measures of spiritual well-being and existential quality of life, and has been hindered by the lack of instruments specifically assessing existential distress and wellbeing. Our aim was to develop a valid and reliable instrument to measure this dimension.

**Methods:**

First, we created a list comprising more than 200 items that address the main existential themes as described by the philosophical and clinical literature. Out of these, 84 were retained after pilot testing and exclusion of the items that showed unsatisfactory psychometric properties. A total of 411 non-clinical participants with a wide range of age groups and educational levels participated in the validation study with a cross-sectional design and a 4-week follow-up. They completed the new instrument, named the 'Existential Dimension Inventory' (EDIN), along with several criterion measures, such as the 15-item Dispositional Resilience Scale, the 18-item Personal Well-Being Scale, the Experiences in Close Relationships Questionnaire, the 18-item Brief Symptom Inventory, the Maudsley Obsessive–Compulsive Inventory, the Authenticity Scale, and the Temperament and Character Inventory. They also completed the EDIN for a second time after 4 weeks. After performing principal component analysis with orthogonal rotation, we estimated the internal consistency and test-retest stability of EDIN factorially derived scales. Convergent and discriminant validity were assessed by examining the correlations between EDIN total and subscale scores, and scores on the criterion measures.

**Results:**

Eight factors (interpreted as Mastery, Fear of loss and death, Authenticity, Serenity in relationships, Pressure of time, Openness to others, Worry about meaning in life, and Loneliness) that accounted for 53% of the total variance were extracted. All EDIN scales displayed high internal consistency and stability, and the pattern of correlations between EDIN scores and their relevant criterion measures was consistent with expectations. Also, the results supported discriminant validity with respect to emotional distress, psychiatric symptomatology, and temperament and character dimensions.

**Conclusion:**

These findings suggested that the EDIN may allow valid and reliable measurement of existential well-being. Many EDIN subscales cover themes identified by existential philosophers and therapists as key issues that human beings face in their everyday lives, which corroborates the relevance of the dimension of existential wellbeing as measured by the EDIN. Despite some limitations, this study supports the validity and reliability of the EDIN. It suggests that this instrument holds the promise of being a valuable tool for research, clinical, and training purposes.

## 1 INTRODUCTION

Though philosophers have practiced existential counselling since ancient times, it is only in the 20^th^ century that such an approach has been formally developed and recognized as a form of psychotherapy. Grounded in existential philosophy, the existential approach to psychotherapy addresses the human condition, the pain inherent in existence, and the way in which human beings live and exist in the world. Its theoretical roots lie in the work of a number of philosophers and writers, such as Sören Kierkegaard, Friedrich Nietzsche, Edmund Husserl, Martin Heidegger, Jean-Paul Sartre, Maurice Merleau-Ponty, Martin Buber, Fyodor Dostoevsky, Karl Jaspers, Paul Tillich, Gabriel Marcel, Simone de Beauvoir, and Albert Camus. Although these authors’ views differ substantially from each other, all of them focus on the actual existence of human beings.

The existential approach to psychotherapy does not present a specific set of procedures or rules for therapy. Rather, it connotes an attitude that proceeds from its philosophical foundations [1]. As the philosophical movement on which existential therapeutic practice is based is so diverse and complex, over the decades, the term ‘existential therapy’ has come to refer to so many different therapeutic approaches that it is impossible to define the field of existential therapy in a single way [1, 2], which makes it more appropriate to refer to ‘existential psychotherapies’ rather than to a single existential psychotherapy [3].

As a matter of fact, a wide range of therapeutic approaches devised in different countries fall under the existential umbrella [4]. In Switzerland, Medard Boss developed the Daseinsanalysis approach that is grounded in Heidegger’s work, which encourages patients to open themselves up to their ‘Being-in-the-world’. In Austria, Victor Frankl developed Logotherapy, or “healing through meaning” in Greek, which places particular emphasis on helping patients find meaning and purpose in their lives. In the United States, authors such as James Bugental, Rollo May, and Irvin Yalom developed a humanistic-existential approach that emphasizes helping patients uncover their subjective experiences and fearlessly face the givens of their existence. In the UK, authors such as Emmy van Deurzen, Ernesto Spinelli, and Hans Cohn developed approaches that shift from diagnosis, pathologization, and value judgments to an exploration of the patient’s lived world and problems in living. In Italy, Luigi De Marchi developed a humanistic-existential approach that integrates several theoretical views and places special emphasis on the embodied nature of human existence, death anguish, and the ensuing existential tension, as well as the therapeutic value of empathy, solidarity, and compassion [5, 6]. Some recently developed short-term approaches developed in palliative care settings, such as group [7] and individual [8] meaning-centered psychotherapy, Managing Cancer and Living Meaningfully (CALM) [9], and dignity therapy [10] may also be numbered among existential therapies.

While existential psychotherapies use a variety of approaches to case conceptualization, therapeutic aims, and intervention strategies, they share several aspects, such as a focus on the patient rather than the symptom, a genuinely empathic therapeutic relationship, a tendency to work with the concrete actuality of patients’ experiences, a general aim to help patients become more authentic, to live more in congruence with their true values and beliefs, to acknowledge their freedom and responsibility, and act upon them, to accept unpleasant feelings, such as anxiety, sadness, guilt and despair, and to learn from them. Creativity, love, authenticity, and free will are valued as potential means of transformation that may help patients live more meaningful lives in the face of uncertainty and suffering [4].

The potential field of application of existential therapies extends beyond definite mental disorders, as their focus on existence may make them particularly suitable for people who have recently experienced an existential crisis, are undergoing significant life changes, are transitioning to a new life stage, are confronting existential questions, have a life-threatening illness, or are close to death. However, as compared with other psychotherapeutic approaches, in spite of their long history, existential therapies are still on the margins of mainstream practice. A key reason for their limited diffusion is the scarcity of research based on traditional methodological approaches in this area, where studies have mostly relied on qualitative methods emphasizing understanding and description. However, influential authors have underscored that the scarcity of research in this field is an issue that needs to be addressed urgently [11], and that practitioners in this tradition do not need to be afraid of quantitative outcome research [12].

A difficulty inherent in carrying out such research is that there is an abundance of measures of psychiatric symptoms, such as depression and anxiety, but a shortage of measures that would be more suitable to detect the most powerful expected beneficial effects of the approach, i.e., those on existential distress and wellbeing. Some studies have used measures of spiritual well-being, which include not only a religious component, but also an existential aspect, which pertains to one’s sense of meaning and purpose in life [13]. Other studies used quality of life measures that include, among others, an ‘existential’ subscale [14, 15]. Other potentially relevant assessment instruments are some measures of affect regulation that were proposed as related to Rogers’ stages of change, as well as measures of real-ideal self-discrepancy, which have parallels with the concepts of congruence and authenticity [16]. Measures of life satisfaction and hedonic well-being have limited overlap with wellbeing in an existential sense, while measures of eudaimonic wellbeing as an orientation towards growth, authenticity, meaning, and excellence [17] bear a greater resemblance, but still pertain to a different construct.

In the light of this scarcity of measures suitable to specifically investigate a properly existential dimension of distress and well-being in clinical settings, we aimed to develop and validate a standardized assessment instrument to measure such a dimension. This paper illustrates the development and validation of a self-completed questionnaire designed for this purpose.

## 2 METHODS

### 2.1 Instrument Development

To develop the new instrument, we followed a commonly used procedure already adopted by one of us in a previous study [18]. First, we designed a large number of items covering the main existential themes, as described by the philosophical and clinical literature. These items describe a wide range of feelings, thoughts, and behaviors that people may experience, with no reference to psychopathological symptoms. In designing the items, we put effort into using language that average people use in everyday situations and avoiding the use of theoretical terms, ambiguous expressions, and abstract or metaphorical language. Each item was scored on a 7-point scale ranging from 1 to 7.

This first list, consisting of more than 200 items, was shown to a group of experienced therapists, who were asked to examine each item for clarity and consistency with existential constructs, and to suggest modifications and further items that could be included. Some items were progressively refined, while other items that were regarded as unclear, ambiguous, or irrelevant by the therapists who examined them were excluded.

This process left 144 items assessing a variety of existential themes, such as illness and death, meaning and purpose in life, losses, authenticity, freedom and autonomy, isolation and loneliness, aging and the passage of time, close relationships, choices, responsibilities, life changes, being recognized and understood, mastery of one’s own life, and openness. This preliminary version of the instrument, that was named the ‘Existential Dimension Inventory’ (EDIN), was subsequently administered to the participants in the validation study.

### 2.2 Validation Study

The preliminary version of the instrument was tested on a large group of participants with a wide range of age and educational levels.

The participants were recruited from the relatives and friends of a group of graduate students, and from people attending a local high school for adults. To be included in the study, a person was required to meet the following criteria: 1) Italian nationality; 2) age 18-65 years; 3) absence of severe medical or psychiatric illness; 4) absence of cognitive impairment. There are no exclusion criteria except those implicit in the inclusion criteria. The study was conducted in accordance with the principles set forth in the Helsinki Declaration. A researcher clearly explained the purpose and procedures of the study to each eligible participant. All participants received a letter describing the study, were given the opportunity to ask questions about the research, and were further required to sign a written informed consent form.

A total of 411 participants completed the preliminary version of the instrument and the other study measures. This sample size is sufficiently large to ensure adequate statistical power for the planned analyses, including factor analysis. Their sociodemographic characteristics are summarized in Table **1**. Subsequently, all participants completed the version of the instrument under evaluation for a second time after 4 weeks.

Together with the version of the instrument that was tested in the study, the participants were given a number of criterion measures to complete, which are described in detail below.

The DRS-15 is a short version of the Dispositional Resilience Scale [19], which is a self-completed instrument that evaluates the construct of psychological hardiness. This questionnaire comprises 15 items, rated on a 4-point scale ranging from 0 (not at all true) to 3 (completely true). Individuals with elevated levels of hardiness have a stronger sense of commitment in life and work, a higher feeling of control, and are more open to change and challenges. They tend to view stressful events and situations as a common part of life that are interesting and valuable. Several studies provided evidence of reliability, criterion-related validity, and predictive validity for the DRS-15 [20, 21].

The 18-item version of the Personal Well-Being Scale (PWB-18) [22] comprises 18 items, scored on a 7-point scale ranging from 1 (“strongly disagree”) to 7 (“strongly agree”). This questionnaire provides scores on six subscales, named self-acceptance (S), positive relationships with others (R), personal growth (G), purpose in life (P), environmental mastery (E), and autonomy (A). People who score high on S have a positive attitude towards themselves, acknowledge and accept diverse aspects of self, including both good and bad facets, and feel good about their past life. Individuals with high scores on R have warm, pleasant, trusting relationships with others, worry about the welfare of others, have a good capacity for empathy, affection, and intimacy, and understand the act of giving and receiving in equal measure that characterizes human relationships. High scorers on G feel they are continually developing, see themselves as growing and flourishing, are open to trying new experiences, sense they are realizing their potential, and feel they are improving over time and are changing consistently with greater self-knowledge and effectiveness. People who score high on P have objectives to achieve in life and a sense of direction, feel that their life is meaningful, and hold convictions that give life purpose. High scorers on M feel able and competent in managing the environment; they control a complex array of external activities, fully take advantage of surrounding opportunities, and are capable of choosing or creating contexts appropriate for their personal needs and values. People who score high on A are autonomous and independent, capable of resisting social pressures to think and act in particular ways; they regulate their behavior from within, and evaluate themselves by their own standards.

The Experiences in Close Relationships (ECR) is a self-report questionnaire to assess adult attachment style, which has established reliability and validity in non-clinical [23], as well as psychiatric [24] samples. The instrument, or shorter scales comprising a number of its items, has been widely used to investigate links between attachment insecurity and a wide range of correlates, including cellular aging [25], immune function [26, 27], brain activity [28] and structure [29], and vulnerability to physical [30, 31] and mental [32] health conditions. The instrument comprises 36 items, each rated on a 7-point scale, and yields scores on two dimensions, named ‘Anxiety’ and ‘Avoidance’, which identify a space on which individual differences in attachment style can be mapped. High scorers on the Anxiety scale tend to be preoccupied with their romantic relationships, to be concerned about insufficient love or abandonment, to have an intense desire for intimacy and closeness, and to ask their partner for more affection and commitment. People who score high on the Avoidance scale feel uneasy with emotional closeness and intimacy, find it difficult to open up to or to depend on their partner, and are reluctant to ask their partner for comfort, advice, or support.

The BSI-18 [33] is a short self-report questionnaire that comprises 18 items taken from the 53-item Brief Symptom Inventory (BSI) [34], which itself is a shortened form of the well-known 90-item Symptom Checklist-90-Revised (SCL-90) [35]. Respondents are asked to rate each item on a 5-point scale according to the severity of the symptom in question during the preceding week. The BSI-18 yields scores on three scales: Somatization, Depression, and Anxiety, each consisting of six items.

The Maudsley Obsessive–Compulsive Inventory (MOCI) [36] is a 30-item self-report questionnaire with a yes/no response format, designed to assess the presence and severity of obsessive-compulsive symptoms. It yields a total score as well as scores on four subscales, named Checking, Washing, Doubting, and Slowness. It is a well-validated instrument [37, 38] that has been used in numerous studies of obsessional symptoms in various populations.

The Authenticity Scale (AS) is a short self-report questionnaire designed to assess an individual’s sense of being authentic [39]. It consists of 12 items scored on a 7-point scale ranging from 1 (does not describe me at all) to 7 (describes me very well). It was developed in accordance with Rogers’s person-centered approach and finds its roots in Barrett-Lennard’s tripartite model of congruence, derived from Rogers’ work [40]. The AS has good psychometric properties and has been widely validated and used [39, 41, 42].

The Temperament and Character Inventory (TCI) is a true/false questionnaire that has been designed to measure temperament and character dimensions according to the psychobiological model of personality, developed by Cloninger and colleagues [43]. This model describes temperament in terms of four prevalently heritable temperament dimensions, named Novelty Seeking (NS), Harm Avoidance (HA), Reward Dependence (RD), and Persistence (P). It conceptualizes character in terms of three dimensions that mature in adulthood, named Self-Directedness (SD), Cooperativeness (C), and Self-Transcendence (ST). We administered the 125-item version of the questionnaire, which has been used by Cloninger himself and his colleagues [44].

### 2.3 Statistical Analyses

All statistical analyses were carried out with SPSS for Mac, version 29.0. All statistical tests were two-tailed, with alpha set at 5%. First, descriptive analyses were performed to examine the distribution of responses to all items of the preliminary version of the EDIN. Then, in order to examine the stability of scores over time, the Intraclass Correlation Coefficient (ICC) between scores on the first and the second administration was calculated for each item. Also, the correlations between scores on each item, and scores on the BSI-18 Depression and Anxiety scales were calculated. No item displayed unsatisfactory test-retest reliability as measured by the ICC or a narrow range of responses, whereas forty-two items with a correlation greater than 0.45 with the severity of depressive or anxiety symptoms were discarded.

Subsequently, exploratory factor analysis was performed on the remaining 102 items in order to examine the factor structure of the EDIN, and to further refine its item composition. The number of factors to be extracted was determined in accordance with the scree-plot method [45]. Orthogonal rotation with the varimax method was performed. As described in detail below, this analysis led to the removal of 15 items that fit poorly with the factorial solution.

A final analysis performed on the remaining 87 items tested the reliability of the factorially derived subscales in terms of internal consistency as measured by coefficient alpha. This led to the elimination of three items that displayed low corrected item-total correlation with their scale. The final version of the instrument, which consists of 84 items, was tested in terms of stability of total and subscale scores between the first and second administration, as measured by the ICC. Finally, the discriminant and convergent validity of the EDIN was tested by examining its correlations with the relevant criterion measures.

First, we examined the relationships between baseline scores on the EDIN and baseline scores on the MOCI, and the BSI-18 Depression and Anxiety scales. A multiple linear regression model was built, in which the EDIN baseline score served as the dependent variable, while gender, age, and baseline scores on the MOCI and the BSI-18 Depression and Anxiety scales were entered as the independent variables. In this analysis, as well as in the other multiple regression analyses, participants with missing data on any variable were excluded from the analysis.

Subsequently, we examined whether changes in the EDIN total score were related to changes in the severity of anxiety, depression, and obsessive-compulsive symptoms. With this aim, change scores (i.e., scores at second administration minus baseline scores) on the EDIN, BSI-18, and MOCI were computed, and a multiple linear regression model was built. In this model, the dependent variable was the change in the EDIN total score, while gender, age, and change scores on the MOCI and the BSI-18 Depression and Anxiety scales were entered as the independent variables.

Subsequently, a multiple linear regression was performed in order to examine the relationships between EDIN scores and temperament and character dimensions. In this regression model, the dependent variable was the EDIN total score, while gender, age, and the TCI scales served as the independent variables.

Also, Pearson’s correlation coefficient was used to examine the relationship between selected EDIN subscales and the relevant criterion measures.

## 3 RESULTS

### 3.1 Factor Structure of the EDIN

The suitability of data for factor analysis was checked with the Kaiser-Meyer-Olkin (KMO) measure of sampling adequacy and Bartlett’s test of sphericity. The first was 0.94 and the second was highly significant (p < 0.001). Both tests indicated that the data were suited for factor analysis. In the eigenvalue plot, we observed a point of inflection after the 8^th^ factor, where the eigenvalue curve flattened out. Thus, eight factors were extracted, which accounted for 53.8% of the total variance. After orthogonal rotation, a fairly simple structure emerged, with a pattern of loadings where most items loaded highly on one and only one factor, with little overlap. Communality values were fairly high, which indicates that the majority of variables fit well within the identified factor structure.

The factor I, interpreted as ‘Mastery’, explained 13.5% of variance after rotation and was loaded with items describing the sense of being able to make decisions, take responsibilities, and face changes in life.

Factor II, interpreted as ‘Fear of loss and death,’ accounted for 9.2% of variance after rotation, and was defined by items indicating fear of severe disease and death, concerning both the respondent and his or her dear ones.

Factor III explained 8.7% of variance after rotation and was interpreted as ‘Authenticity’ since it was characterized by items referring to the sense of being authentic, free, and not conditioned by other people.

Factor IV explained 5.4% of variance after rotation and was interpreted as ‘Serenity in relationships,’ since it was defined by items that reflect satisfaction with one’s own intimate relationship and sense of serenity in love.

Factor V, interpreted as ‘Pressure of time,’ explained 5.3% of variance after rotation and was characterized by items describing sense of time ticking fast and worry about aging.

Factor VI explained 4.8% of variance after rotation and was interpreted as ‘Openness to others’ since it was loaded by items indicating being comfortable with people holding different opinions or belonging to different cultures.

Factor VII, interpreted as ‘Worry about meaning in life,’ explained 4.3% of variance after rotation, and was defined by items describing reflections on the meaning and purpose of life.

Finally, Factor VIII explained 2.5% of variance after rotation, and was interpreted as ‘Loneliness,’ since it was characterized by items referring to feelings of loneliness and worry about being abandoned by loved ones.

Subsequently, the items were further selected in two ways. First, 15 items that did not fit well with the factor solution were removed: one item did not load more than 0.32 (10% shared variance between variable and factor) on any factor; six items showed loadings of similar size on two factors; for other eight items, their content was inconsistent with the factor they loaded on, and did not contribute to the interpretation of the factor. Second, three items were discarded after a preliminary reliability analysis, as they displayed a corrected item-total correlation with their scale that was so low that their removal would have led to an increase in the internal consistency of the scale as measured by coefficient alpha.

### 3.2 Reliability of the Scales

The ‘Mastery’ scale (25 items) displayed high reliability; internal consistency as expressed by coefficient alpha was 0.95 and test-retest reliability as measured by the ICC was 0.95 (95% C.I. 0.94-0.96). Greater scores indicate a higher sense of self-confidence and mastery of one’s own life.

The ‘Fear of loss and death’ scale (13 items) also displayed high reliability, with a coefficient alpha of 0.92 and an ICC of 0.93 (95% C.I. 0.91-0.94). Higher scores indicate greater fear of falling ill, dying, and losing dear ones.

The ‘Authenticity’ scale (11 items) showed similarly high reliability; the coefficient alpha was 0.91, and the ICC was 0.93 (95% C.I. 0.92-0.94). Higher scores reflect a greater sense of being true to oneself and free from external influences.

The ‘Serenity in relationships’ scale (7 items) was also found to be highly reliable, with a coefficient alpha of 0.89 and an ICC of 0.93 (95% C.I. 0.92-0.94); higher scores indicate greater serenity in love.

The ‘Pressure of time’ scale (10 items) showed high reliability, too, as indicated by a coefficient alpha of 0.91 and an ICC of 0.94 (95% C.I. 0.93-0.95). Higher scores reflect greater feelings of time running fast and of worry about aging.

The ‘Openness to others’ scale (8 items) also displayed high reliability, with a coefficient alpha of 0.86 and an ICC of 0.92 (95% C.I. 0.91-0.94); higher scores indicate greater acceptance of different opinions and cultures.

The ‘Worry about meaning in life’ scale (7 items) displayed similarly high reliability; the coefficient alpha was 0.85, and the ICC was 0.93 (95% C.I. 0.92-0.94). Higher scores reflect greater worry about living a meaningful life.

Satisfactory reliability coefficients were also found for the ‘Loneliness’ scale (3 items), as indicated by a coefficient alpha of 0.80 and an ICC of 0.92 (95% C.I. 0.91-0.93); higher scores indicate greater feelings of loneliness and fear of being alone.

As far as the total score of Existential well-being is concerned, in its calculation, the ‘Fear of loss and death’, ‘Pressure of time’, ‘Worry about meaning in life’, and ‘Loneliness’ scales are reverse-keyed so that higher total scores indicate greater existential wellbeing. The total score displayed very high reliability, with a coefficient alpha of 0.97 and an ICC of 0.94 (95% C.I. 0.93-0.95).

### 3.3 Criterion-related Validity

Table (**2**) shows the results of the multiple regression analysis investigating the relationship between EDIN total score and gender, age, MOCI, and BSI-18 Depression and Anxiety scores. The table reports R, R^2^, adjusted R^2^, the zero-order correlations, the standardized regression coefficients (Beta), and the squared semipartial correlations, which represent the proportion of variance in the dependent variable, that is uniquely explained by the independent variable to which it refers. One participant was excluded from this analysis due to missing data.

Table (**3**) summarizes the results of the multiple regression analysis investigating the relationship between change in the EDIN total score and change in MOCI and BSI-18 Depression and Anxiety scores. Five participants with missing data were excluded from this analysis.

Table (**4**) shows the results of the analyses focusing on the relationship between the EDIN total score and TCI scores. Three participants were excluded from this analysis due to missing data.

Concerning the relationship between the selected EDIN subscale scores and their relevant criterion measures, the pattern of correlations was consistent with expectations. In particular, the Mastery scale was positively correlated with the TCI Self-Directedness scale (r=0.63), the PWB Environmental mastery scale (r=0.61), and the DRS total score (r=0.57); the Openness to others scale was positively correlated with the TCI Cooperativeness scale (r=0.48); the Serenity in relationships scale was positively correlated with the PWB Positive relationships with others scale (r=0.42) and negatively correlated with the ECR Avoidance (r = -0.56) and Anxiety (r = -0.42) scales; the Loneliness scale was positively correlated with the ECR Anxiety scale (r=0.48); the Worry about meaning in life scale was negatively correlated with the PWB Purpose in life scale (r = -0.24); the Authenticity scale was positively correlated with the PWB Autonomy (r=0.47) and Self-acceptance (r=0.61) scales and with the MSA Authentic living scale (r=0.58) and negatively correlated with the MSA Acceptance of external influences (r = - 0.59) and Self-alienation (r = -0.72) scales. All these correlations are statistically significant (p<0.001).

## 4 DISCUSSION

In this paper, we described the development and validation of a self-report instrument designed to measure the construct of existential well-being, which is central in psychotherapy approaches derived from humanistic and existentialist philosophy. After a lengthy process of construction and refinement of a self-completed questionnaire named ‘Existential Dimension Inventory’ (EDIN), which inquires about thoughts, feelings and actions related to crucial existential domains with no reference to psychopathological symptoms, we administered it to a large group of non-clinical participants distributed across a wide range of age groups and education levels. We identified a number of dimensions underlying existential wellbeing, which we called ‘Mastery’, ‘Fear of loss and death’, ‘Authenticity’, ‘Serenity in relationships’, ‘Pressure of time’, ‘Openness to others’, ‘Worry about meaning in life’, and ‘Loneliness’.

After further item selection on the grounds of the results of factor analysis and classical psychometric theory, the factorially derived scales of this self-report questionnaire displayed high reliability in terms of both internal consistency and stability of scores over time. Also, the meaningful correlations observed between the EDIN scales and their relevant criterion measures provided evidence of convergent validity. Furthermore, the small statistical relationship between the EDIN total score and the severity of anxious and obsessive-compulsive symptoms, the small to moderate relationship between the EDIN total score and the severity of depressive symptoms, and the small relationship between the changes in the EDIN total score over a 4-week period and the changes in the severity of anxious, obsessive-compulsive, and depressive symptoms over the same period support discriminant validity with respect to emotional distress and psychiatric symptomatology. While we observed a modest degree of overlap between existential wellbeing as measured by the EDIN and depressive symptoms, it should be noted that some degree of overlap is expected between depression and any construct pertaining in some way to psychological distress or emotional suffering. For instance, not only anxiety and depression diagnoses tend to co-occur, but their symptoms are highly correlated, with numerous studies reporting large covariance between depressive and anxiety symptoms in clinical as well as non-clinical samples [46, 47]. Even established trait variables that are clearly separate from depression, such as neuroticism, extraversion [48], and alexithymia [49] display a substantial degree of correlation, either positive or negative, with depression. Likely reasons that may account for the modest degree of association between depression and lower levels of a construct, such as existential wellbeing, which correspond to existential distress, are shared variance with broader constructs such as negative affect or emotional wellbeing, and some degree of similarity between certain symptoms of depression, such as feeling alone and difficulty making decisions, and some facets of existential wellbeing, such as higher Loneliness and lower Mastery.

Support for discriminant validity is also provided by the finding that only 18% of the variance in the EDIN total score was uniquely accounted for by temperament and character dimensions. These findings corroborate the notion that the construct of existential wellbeing, as measured by EDIN, is distinct from the mere concept of low levels of emotional distress and from major personality constructs.

These findings suggest that the EDIN self-report instrument may allow valid and reliable measurement of existential well-being. Given that the sample was purposely selected to include not only young and well-educated people but rather participants with a wide range of age groups and educational levels, the study findings should be fairly generalizable.

The relevance of the dimension of existential wellbeing as measured by the EDIN for clinical practice is corroborated by the observation that many of the instrument subscales cover a number of themes identified by existential philosophers and therapists as key issues that human beings experience in their day-to-day lives.

The themes covered by the Mastery subscale, such as the sense of being able to make decisions, take responsibilities, and face changes in life share many similarities with the emphasis of the existentialist literature on adopting a decisive attitude toward existence, being committed to personal meaning and objectives, and taking responsibility towards oneself and one’s own life in order to be able to make deliberate choices and transcend the immediate circumstances of life [4, 50, 51]. Indeed, given that in the existentialist perspective there is no pre-existing essence that precedes and determines existence [52, 53], being able to make choices, and take responsibility for them is absolutely crucial as individuals make their own identity through their choices and actions. Also, given that in the existentialist perspective there is no moral imperative, divine will, or natural law that can guide our decisions or justify them, it is important to be able to cope with the anxiety that comes from realizing that we alone are responsible for our choices and our actions, in order to act in the face of the uncertainty inherent in the human condition and to take responsibility for wherever our actions might lead [54-56]. Kierkegaard [57] emphasized the tension between freedom and human existence linking anxiety, which he regarded as a necessary condition for human choice and responsibility, to freedom. Although freedom entails anxiety, the possibility of attaining freedom arises through facing anxiety. Higher scores on the Mastery subscale suggest that the respondent is capable of taking responsibilities and making choices in the face of this existential tension, and thus indicate greater existential wellbeing.

The fear of severe disease and death captured by the Fear of Loss and Death subscale, and the sense of time ticking fast and worry about aging tapped by the Pressure of Time subscale resonate with a pivotal theme of existential philosophy, as human life is put into perspective by death and temporality. On one hand, the awareness of one’s own death and the uncertainty about the time of its occurrence elicits a certain degree of anxiety. On the other hand, it brings an awareness of life which drives attention from trivial preoccupations towards things that really matter and promotes active exploration of existential issues in life [52, 58-60]. In particular, the items composing the Fear of Loss and Death subscale, and the items relating to worry about aging of the Pressure of Time subscale indicate an attitude toward one’s own life that is a characteristic of what Heidegger would call an inauthentic way of Being, where people see the phenomenon of death as something that constantly occurs in the world, as an abstract, an anonymous event that happens to others. On the contrary, an authentic way of being implies facing one’s own death, confronting the anxiety that arises from the understanding of one’s own finitude, and leverage this anxiety to grasp one’s own existential freedom and possibilities [52]. High scores on these two subscales suggest that the respondent is still in the midst of this process, and has still not come to terms with his or her aging and death; therefore, the scales are reverse-keyed as high scores indicate lower existential wellbeing.

The sense of being authentic, free, and not conditioned by other people covered by the Authenticity subscale has much in common with another central theme of existential philosophy, that views living an authentic life as a pillar of healthy functioning. Existentialism, indeed, is highly critical of mass society and of people’s tendency to conform to the norms and expectations of the public. This criticism stems from the belief that although a conformist way of living can be comforting, it is also a manifestation of inauthenticity. For instance, Kierkegaard described inauthenticity in terms of fleeing from ourselves, and cautioned against living a life where we let others decide our lives for us, and we are unable to make any real commitment [61]. In a similar vein, Heidegger referred to this condition as a form of estrangement where one exists as a “they-self” that just goes with the tide [52]. Authentic living is about being true to oneself, seeing one’s own life and oneself in an honest way, and accepting the limitations of life as well as one’s own personal limitations. It involves recognizing one’s own freedom and responsibility for oneself, and it brings with it openness both to oneself and to others [4, 51, 58, 62]. Indeed, an authentic existence involves having the courage to embrace the uncertainty of the future, to bear the anguish inherent in the human condition, and to become a “being towards death” [52], because accepting one’s own mortality as a real possibility is key to living authentically. Also, as underscored by Kierkegaard, authenticity requires great effort and commitment because being true to oneself cannot be achieved merely by following some universal principles. The subjective truth of the individual fills the holes that cannot be covered by objective truth, is necessary to have a full idea of what counts as personal truth, and is higher than truths grounded in social or moral norms [63, 64]. Consequently, higher scores on the Authenticity subscale indicate greater existential wellbeing.

The existential ideas of authenticity and freedom are also linked to feeling at ease with individuals who have different opinions or come from diverse cultures, a perspective captured by the Openness to Others subscale. Existentialism underscores that there is an ethical responsibility rooted in freedom, as freedom implies the obligation to help others realize their own freedom. Several authors argue that one should resist the temptation to see other people as things to be manipulated and controlled for one’s own use, and to cultivate an authentic relation that involves the mutual recognition of two freedoms [52, 65]. Viktor Frankl, too, observed that pursuing activities in support of others may enable people to transcend suffering even in the most adverse conditions [66]. Therefore, higher scores on this subscale indicate greater existential wellbeing.

The feelings of satisfaction with one’s intimate relationship and serenity in love tapped by the Serenity in Relationships subscale and, conversely, the feelings of loneliness and worry about being abandoned by loved ones covered by the Loneliness subscale both seem to pertain to the *Mitwelt* basic dimension of human existence described by Binswanger [67], which represents our relationships with other human beings and revolves around poles, such as intimacy and isolation, feelings of being loved and of being rejected. While, as underscored by Kierkegaard [57], feelings of anxiety and loneliness are bound to arise over the course of an authentic life that does not rely on the comforting truths of the crowd or its generic conceptions of right and wrong, enduring and pervasive feelings of loneliness indicate lower, rather than greater, existential wellbeing, so the Loneliness subscale is reverse-keyed. On the contrary, higher scores on the Serenity in Relationships subscale suggest greater existential well-being through the development of the capacity for selfless love and interdependence and the achievement of an authentic loving relationship characterized by the reciprocal acknowledgement of the other’s freedom and transcendence [65]. The relevance of a positive experience of love for existential well-being has also been underscored by May, who considered mature love as a process involving mutual respect and understanding [68]. Frankl, too, mentioned the experience of love among the ways through which the creation and discovery of meaning in one’s life can be achieved [69] and underscored that love implies living the experience of another person in all his uniqueness and singularity [69, 70].

Finally, the concern with meaning and purpose in life covered by the Worry about meaning in life subscale strongly relates to other pivotal concerns rooted in human existence. Indeed, the topic of meaning is ubiquitous in the existentialist literature. For instance, Heidegger suggested that the mode of being in the world that is particular to human beings is a meaning-giving activity. In his view, meaning is not the product of detached cognitive operations, but rather it emerges against the background of people’s functional involvement in the world [52]. Viktor Frankl, the author who probably put the greatest emphasis on the topic of meaning, held that finding meaning or purpose in life is the primary motivational force for individuals and that human beings have a basic need to engage in something that gives their life a purpose. He emphasized that life can have a purpose even in the face of suffering, and that people can find meaning through their attitudes, choices, and actions [69]. In his view, each individual solely decides the meaning of his or her life, and he or she has to take responsibility for creating and deciding its unique meaning. Among contemporary authors, Van Deurzen described the spiritual dimension or *Überwelt* [71] where people find meaning by assembling all the pieces of the puzzle together for themselves, while facing the contradictions connected with the tension between purpose and absurdity, hope and desperation. Given that higher scores on the Worry about meaning in life subscale indicate greater preoccupation with finding meaning and purpose in existence, rather than the sense of living a meaningful and purposeful life, this subscale is reverse-keyed.

It is worth observing that, while all these factors are clearly relevant from an existential perspective, some of them are of particular relevance in the current cultural and social context. The digital revolution is having an undeniable impact on the human brain and behavior, as people now dedicate a significant portion of their time to digital environments [72]. Digital media, most of all the internet, have become important aspects of our life, and they carry risks as well as advantages. Social isolation and reduced real-life interactions are among the risks linked to excessive use of the internet [73-75], and this makes the relevance of the Loneliness subscale greater than ever. The Authenticity subscale is particularly relevant, too, in this regard, as it is reasonable to presume that the widespread use of digital technologies, particularly social networks, online chat platforms, forums, and other virtual spaces, is making it more difficult for people, especially youngsters, to live an authentic life, given that they are becoming increasingly and readily exposed to the opinions and attitudes of a large number of peers, which may lead to greater conformity among individuals. Also, both the Mastery and the Worry about meaning in life subscales, with their strong links to the topics of freedom and choice, bear particular relevance in an age where issues such as pandemic-related social and economic changes, climate change, information overload, and misinformation are probably leaving people more confused than ever. The troubling questions of freedom and the meaning of one’s own existence are even more pressing in an era of increased uncertainty about where to look for a firm and steady sense of truth and meaning [76].

On one hand, a number of the dimensions underlying existential wellbeing as measured by the EDIN questionnaire share similarities with the constructs measured by other assessment instruments, such as emotional distress, anxiety, depression, hedonic and eudaimonic wellbeing, life satisfaction, spirituality, spiritual wellbeing, and quality of life. On the other hand, the results of convergent and discriminant validity analysis suggest that the existential dimension defined by the EDIN items is not redundant with these constructs, either singularly or as a whole. This is consistent with the theoretical framework that guided the development of the instrument, which was firmly grounded in the work of existential philosophers and therapists.

Some limitations of this study should be acknowledged. While we assessed discriminant validity against one of the most influential models in personality theory, we did not measure other prominent personality models, such as the ‘Big Five’ factors model. Therefore, we cannot completely exclude a substantial overlap between the construct of existential wellbeing as measured by the EDIN and major personality constructs. Future studies should be able to address this issue by including a measure of the Big Five factors and examining the degree of overlap with EDIN scores. Until then, some caution should be warranted in interpreting the results of research conducted with the EDIN, as the possible influence of personality factors, such as neuroticism, cannot be ruled out at the moment. Second, we did not examine the instrument responsivity to clinically meaningful change, which would have involved the study of people with clinically significant emotional distress tested before and after a therapeutic intervention. However, such a study of the clinical properties of an instrument conceptually pertains to subsequent phases of its development, and future studies would allow to investigate this aspect. Indeed, studies on clinical populations are undoubtedly needed not only to investigate the issue of responsiveness, which is a crucial prerequisite for the use of the EDIN as an outcome measure in clinical research, but also to extend the validation of the instrument beyond non-clinical participants to people suffering from mental disorders or experiencing an existential crisis. We definitely plan to perform such a study in the near future. It should also be kept in mind that the object of measurement is a rather narrow construct of wellbeing, defined from the perspective of existential and humanistic psychology, rather than the wider concepts of hedonic and eudaimonic wellbeing that are quite established in the psychological literature. It should also be acknowledged that the EDIN, as all self-report instruments, can assess only conscious processes accessible to introspection. Further, it cannot claim to measure all that is relevant to the concept of existential wellbeing, but only the topics that are covered by its items. While the instrument can permit a valid and reliable assessment of the existential dimension, it cannot substitute for an in-depth clinical assessment individualized for each patient, which remains mandatory. Nevertheless, it allows a standardized evaluation of a number of the main existential themes and of many issues everyone is concerned about.

## CONCLUSION

In conclusion, the satisfactory psychometric properties displayed by the EDIN questionnaire suggest that an existential well-being dimension, theoretically rooted in the work of existential philosophers and humanistic approaches to psychotherapy, can be validly and reliably measured. It is reasonable to suggest that the EDIN instrument could play a valuable role in the initial evaluation of individuals preparing to undergo mental health treatment—not as a replacement for established assessment tools, but as a complementary resource that may offer clinicians deeper insight into the patient. For existential therapists, the instrument might be useful not only to gauge the level of existential distress in their patients, but also to identify where the patient’s difficulties lie *via* a quick review of the responses, and how he or she responds to the givens of life. It can also prove to be useful in measuring the level of existential wellbeing in non-clinical populations and possibly to detect people with a high level of distress who need attention and treatment. However, this latter, potential use of the EDIN requires further study to determine if there is a valid cut-off score to identify people in need. Another important potential use of the EDIN lies in the research field, as it would allow the measurement of a dimension that is a key focus of humanistic and existential approaches to psychotherapy. Finally, the EDIN questionnaire could be useful in the training of psychotherapy trainees, and of residents in clinical psychology and psychiatry, as it may help them to get familiarized with humanistic and existential concepts and constructs, and to include them in their theoretical background.

## Figures and Tables

**Table 1:** Demographic characteristics of participants

	**N (%)**	**Mean (SD)**
**Sex**		
Male	204 (49.6)	
Female	207 (50.4)	
**Age**		39.8 (13.2)
**Education**		
Primary school	7 (1.7)	
Junior high school	88 (21.4)	
Senior high school	139 (33.8)	
University degree	177 (43.1)	
**Marital status**		
Unmarried	187 (45.5)	
Living with a partner	56 (13.6)	
Married	132 (32.1)	
Separated or divorced	27 (6.6)	
Widowed	9 (2.2)	

**Table 2:** Multiple regression analysis with EDIN total score as dependent variable and gender, age, MOCI scores, and BSI-18 Depression and Anxiety scores as independent variables.

-	EDIN total score		
**F**	52.5*** (df 5, 404)		
**R**	0.62		
**R^2^**	0.39		
**Adjusted R^2^**	0.38		
**Gender** **(female=0, male=1)**	**r**	**β**	**sr^2^**
	0.19 **	0.07	0.005
**Age**	0.05	-0.08 *	0.006
**MOCI**	-0.41 ***	-0.22 ***	0.040
**BSI-18 Depression**	-0.57 ***	-0.38 ***	0.063
**BSI-18 Anxiety**	-0.52 ***	-0.15 **	0.009

* p ≤0.05

** p ≤0.01

*** p ≤0.001

r = zero-order correlation

β = standardized regression coefficient

sr^2^ = squared semipartial correlation

**Table 3:** Multiple regression analysis with change in the EDIN total score as dependent variable, and gender, age, and change in MOCI scores and in BSI-18 Depression and Anxiety scores as independent variables.

-	**Change in EDIN Total Score**		
**F**	16.3*** (df 5, 400)		
**R**	0.41		
**R^2^**	0.17		
**Adjusted R^2^**	0.16		
**Gender** **(female=0, male=1)**	**r**	**β**	**sr^2^**
	0.05	0.04	0.002
**Age**	-0.05	-0.05	0.002
**Change in MOCI**	-0.31 ***	-0.26 ***	0.060
**Change in BSI-18 Depression**	-0.32 ***	-0.32 ***	0.065
**Change in BSI-18 Anxiety**	-0.15 **	0.13 *	0.010

* p ≤0.05

** p ≤0.01

*** p ≤0.001

r = zero-order correlation

β = standardized regression coefficient

sr^2^ = squared semipartial correlation

**Table 4:** Multiple regression analysis with EDIN-20 total score as dependent variable and gender, age, and TCI scores as independent variables.

-	**EDIN total score**		
**F**	50.4*** (df 9, 398)		
**R**	0.73		
**R^2^**	0.53		
**Adjusted R^2^**	0.52		
**Gender** **(female=0, male=1)**	**r**	**β**	**sr^2^**
	0.19 **	0.06	0.002
**Age**	0.05	-0.05	0.002
**Novelty Seeking**	-0.03	-0.02	0.0002
**Harm Avoidance**	-0.58 ***	-0.33 ***	0.061
**Reward Dependence**	-0.15 **	-0.07	0.004
**Persistence**	0.06	-0.01	0.0001
**Self-Directedness**	0.66 ***	0.45 ***	0.111
**Cooperativeness**	0.31 ***	0.06	0.003
**Self-Transcendence**	-0.06	-0.04	0.002

* p ≤0.05

** p ≤0.01

*** p ≤0.001

r = zero-order correlation

β = standardized regression coefficient

sr^2^ = squared semipartial correlation

## Data Availability

The data are not publicly available due to legal restrictions according to Italian legislation, as they contain information that could compromise the privacy of research participants within the article.

## LIST OF ABBREVIATIONS

CALM: = Cancer and Living Meaningfully
EDIN: = Existential Dimension Inventory
ECR: = Experiences in Close Relationships
BSI: = Brief Symptom Inventory
SCL-90: = Symptom Checklist-90
MOCI: = Maudsley Obsessive–Compulsive Inventory
AS: = Authenticity Scale
NS: = Novelty Seeking
ICC: = Correlation Coefficient
KMO: = Kaiser-Meyer-Olkin

## STANDARDS OF REPORTING

STROBE guidelines were followed.
